# Reviewer acknowledgement 2013

**DOI:** 10.1186/1471-244X-14-40

**Published:** 2014-02-13

**Authors:** Alice K Murray

**Affiliations:** 1 BioMed Central, 236 Gray’s Inn, Road, London WC1X 8HB, UK

## Abstract

**Contributing reviewers:**

The editors of BMC Psychiatry would like to thank all our reviewers who have contributed to the journal in Volume 13 (2013).

## 

Dag Aarsland

Norway

Giovanni Abbate-Daga

Italy

Amelie Achim

Canada

Sophia Adams

Australia

Ana Adan

Spain

Mats Adler

Sweden

Gwen Adshead

United Kingdom

Miroslav Adzic

Serbia

Christopher Ahnallen

USA

Kirsi Ahola

Finland

Martin Aigner

Austria

Emine Aksoydan

Turkey

Martin Alda

Canada

Carlo Altamura

Italy

Antonio Alvim-Soares

Brazil

Federico Amianto

Italy

Marija Anderluh

Slovenia

Gerhard Andersson

Sweden

Kurt Angstman

USA

Niki Antypa

Italy

Paul Appelbaum

USA

Guro Årdal

Norway

Cynthia Arfken

USA

C.P. Arun

United Kingdom

Philip J Asherson

United Kingdom

Majed Ashy

USA

Sally Askey-Jones

United Kingdom

Anu Asnaani

USA

Carlos Ayan

Spain

Touraj Ayazi

Norway

Jose Luis Ayuso-Mateos

Spain

DUSAn Backovic

Serbia

Christopher Baethge

Germany

Fatemeh Bahrami

Iran

Ursula Franziska Bailer

USA

Ursula Franziska Bailer

Austria

Maarten Bak

Netherlands

Dewleen Baker

USA

Jessica Baker

USA

John Baker

United Kingdom

Jacob Ballon

USA

Wojciech Baranowski

Poland

Izabela Barbosa

Brazil

Rachel Barnes

USA

Fiona Barnett

Australia

Anna Bartak

Netherlands

Ankur Barua

Nepal

Daniel Bateman

USA

Philip Batterham

Australia

Mark Bauer

USA

Harald Baumeister

Germany

Karl Bechter

Germany

Massimiliano Beghi

Italy

Ina Beintner

Germany

Rita Bella

Italy

Sarah Bendall

Australia

Corina Benjet

Mexico

Basil Bereza

Canada

Marcelo Berlim

Canada

Miguel Bernardo

Spain

Elizabeth Berry-Kravis

USA

Sylvie Berthoz

France

Daniel Bessesen

USA

Kamaldeep Bhui

United Kingdom

Bruno Biancosino

Italy

Barbara Bien

Poland

Patricia Bijttebier

Belgium

Andreas Birgegård

Sweden

Istvan Bitter

Hungary

Edward Bixler

USA

Mari Asphjell Bjornaas

Norway

Hilario Blasco-Fontecilla

Spain

Marc B.J. Blom

Netherlands

Sune Bo

Denmark

Petr Bob

Czech Republic

Claudi Bockting

Netherlands

Stefania Bonaccorso

United Kingdom

Paola Bortolaso

Italy

Lynn Boschloo

Netherlands

Judith Bosmans

Netherlands

Vladimir Bostanov

Germany

Rami Bou Khalil

Lebanon

Carole Boudebesse

France

Corin Bourne

United Kingdom

Martine Bouvard

France

Charles Bowden

USA

Lindy-Lou Boyette

Netherlands

Francesca Brambilla

Italy

Anke Bramesfeld

Germany

Jorgen Bramness

Norway

Serge Brand

Switzerland

Eva Brandl

Canada

Keith Bredemeier

USA

Alfiee Breland-Noble

USA

Avril Brereton

Australia

Damien Brevers

Belgium

Warrick Brewer

Australia

William Brinkman

USA

Timo Brockmeyer

Germany

Evelien Brouwers

Netherlands

Amanda Joelle Brown

USA

Traolach Brugha

United Kingdom

Scott Brunero

Australia

Tom Bschor

Germany

Reiner Buchhorn

Germany

Benjamin Buck

USA

Kelly Buck

USA

Oscar Bukstein

USA

Antonio Bulbena

Spain

Andrew Bulloch

Canada

Robertas Bunevicius

Lithuania

Massimiliano Buoli

Italy

Christoph Burkhart

Switzerland

Peter Byrne

United Kingdom

Lionel Cailhol

Canada

Eve Caligor

USA

James Cantor

Canada

Christy Capone

USA

Vladimir Carli

Sweden

Natacha Carragher

Australia

Mauro Giovanni Carta

Italy

Greg Carter

Australia

Patricia Casey

Ireland

David Castle

Australia

Kate Cavanagh

United Kingdom

Ausiàs Cebolla

Spain

Matteo Cella

United Kingdom

Rolieke Cents

Netherlands

Linda Chafetz

USA

Maud Champagne-Lavau

France

Lai Fong Chan

Malaysia

Chein-I Chang

USA

Kristine Chapleau

USA

Alice Charach

Canada

Fiona Charlson

Australia

Sudipto Chatterjee

India

Helen Chen

Singapore

Lian-Yu Chen

USA

Yougen Cheng

China

Manoranjenni Chetty

United Kingdom

Lin-Yang Chi

Taiwan

I-Chia Chien

Taiwan

Marco Chiesa

United Kingdom

Luigi Rocco Chiri

Italy

Jimmy Choi

USA

Jiyeon Choi

USA

Arabinda Narayan Chowdhury

United Kingdom

Pierre Chue

Canada

Lukasz Cichocki

Poland

Bjorgulf Claussen

Norway

Elise Clerkin

USA

Kerri Clough-Gorr

USA

Alex Cohen

United Kingdom

Amy Cohen

USA

Ian Colman

Canada

Hannie Comijs

Netherlands

Christine Conelea

USA

Philippe Conus

Switzerland

Cesare Cornaggia

Italy

Bert Cornelius

Netherlands

Samuele Cortese

USA

Marco Cosentino

Italy

Mary-Anne Cotton

United Kingdom

Jennifer Couturier

Canada

Nancy Covell

USA

Peter Alan Coventry

United Kingdom

Luca Cravello

Italy

Mike J Crawford

United Kingdom

Kenneth Critchfield

USA

Anselm Crombach

Germany

Marie Crowe

New Zealand

Gary Cuddeback

USA

Kathryn Cullen

USA

Jill Cyranowski

USA

Riccardo Dalle Grave

Italy

Alec Davidson

USA

Kate Davidson

United Kingdom

Larry Davidson

USA

Darlene Davis

USA

Louanne Davis

USA

Robert Dawe

USA

Domenico De Berardis

Italy

Reny De Leeuw

USA

Vincenzo De Luca

Canada

Jesus De Pedro-Cuesta

Spain

Yves De Roten

Switzerland

Varuni De Silva

Sri Lanka

Michiel De Vries Robbé

Netherlands

Blake Dear

Australia

Tisha Deen

USA

Daniel Deheinzelin

Brazil

Renate Deinzer

Germany

Jakob Demant

Denmark

Ellen Dennehy

USA

Amare Deribew

Ethiopia

Alain Dervaux

France

Claudius Diez

Germany

Giancarlo Dimaggio

Italy

Kylie Dingwall

Australia

Breno Diniz

Brazil

Andreas Dinkel

Germany

Sandra Dittmann

Germany

Tonya Dodge

USA

Hirokazu Doi

Japan

Tara Donker

Australia

Liesje Donkin

New Zealand

Liesje Donkin

Netherlands

Sarah Doucette

Canada

Francois Ducray

France

Anne Duffy

Canada

Vikas Duvvuri

USA

Alma Dzubur - Kulenovic

Bosnia And Herzegovina

Klaus P. Ebmeier

United Kingdom

Cindy Eckart

Germany

Marina Economou

Greece

Eamonn Eeles

Australia

Daryl Efron

Australia

Thomas Ehring

Germany

Thomas Ehring

Netherlands

Stefan Ehrlich

Germany

Solvig Ekblad

Sweden

Oivind Ekeberg

Norway

David Ekers

United Kingdom

Maha El Gaafary

Egypt

Ian Ellison-Wright

United Kingdom

Ulf Engqvist

Sweden

Irene Eriksson

Sweden

Verena Ertl

Germany

Carolina Escobar

Mexico

Jonathan Espie

United Kingdom

Ann Faerden

Norway

Kate Fairweather-Schmidt

Australia

Alessandra Falco

Italy

Xiang Fan

USA

Douglas Faries

USA

Paul Farrand

United Kingdom

Louise Farrer

Australia

Secondo Fassino

Italy

Victor Faundez

USA

Angela Favaro

Italy

Seena Fazel

United Kingdom

Adriana Feder

USA

Bernardino Fernández Calvo

Spain

Fernando Fernandez-Aranda

Spain

Madelyn Fernstrom

USA

Silvia Ferrari

Italy

Anne-Kathrin Fett

Netherlands

Manfred Fichter

Germany

Jess Fiedorowicz

USA

Mario Fioravanti

Italy

Joanna Fiszdon

USA

Kathleen Kara Fitzpatrick

USA

SUSAn Fletcher

Australia

Christoph Flückiger

Switzerland

Andrew Forrester

United Kingdom

Yvonne Forsell

Sweden

Ian Frampton

United Kingdom

Clement Francois

France

Erica Frank

Canada

Ingmar Franken

Namibia

Thomas Frazier

USA

Ann Fridner

Sweden

Ronna Fried

USA

Hans-Christoph Friederich

Germany

Yoshiharu Fukuda

Japan

Sadaaki Fukui

USA

Yasuko Funabiki

Japan

Toshiaki Furukawa

Japan

Suzanne Gag1

United Kingdom

Raphael Gaillard

France

Christopher Gale

New Zealand

Rohan Ganguli

Canada

Carlos Garcia Forero

Spain

Javier Garcia-Campayo

Spain

Carmen Garcia-Pena

Mexico

David Gard

USA

William Gardner

USA

Elena Garralda

United Kingdom

Andrew Garratt

Norway

Santino Gaudio

Italy

Stefan Gazdzinski

Poland

Adam Gerace

Australia

Marloes Gerrits

Netherlands

Elliot S Gershon

USA

Lucio Ghio

Italy

Roger Gibson

Jamaica

Erik Giltay

Netherlands

Ragy Girgis

USA

Elke Gizewski

Germany

Melissa Gladstone

United Kingdom

Heide Glaesmer

Germany

Nathalie Godart

France

Robert Göder

Germany

Terry Goldberg

USA

Daniela Goncalves

United Kingdom

Laura Goodwin

United Kingdom

Marc Gordon

USA

Hans Grabe

Germany

Carla Maria Gramaglia

Italy

Gesine Grande

Germany

Elaine Green

United Kingdom

Tracy Greer

USA

Patricia Groleau

Canada

Sandeep Grover

India

Adam Guastella

Australia

Cristiana Guetti

Italy

Bahar Güntekin

Turkey

Oye Gureje

Nigeria

Manuel Gurpegui

Spain

Peter Gutierrez

USA

Jose Gutierrez-Maldonado

Spain

Cassidy Gutner

USA

Mark Haddad

United Kingdom

Anders Hakansson

Sweden

Seiji Hama

Japan

Jay Hamm

USA

Changsu Han

South Korea

David Hanauer

USA

Brenda Happell

Australia

Catherine Harrington

USA

Carol Harvey

Australia

Philippe-Olivier Harvey

Canada

Robin Harwood

USA

Alkomiet Hasan

Germany

Hossein Hassanian-Moghaddam

Iran

Angela Hassiotis

United Kingdom

Ilanit Hasson-Ohayon

Israel

Stephani Hatch

United Kingdom

SUSAn Hatters Friedman

New Zealand

Jari Haukka

Finland

Lea Hautala

Finland

Phillipa Hay

Australia

Naoki Hayashi

Japan

Dave Hayes

Canada

Marie Hayes

USA

Ulrich Hegerl

Germany

Claire Henderson

United Kingdom

Urs Hepp

Switzerland

Stephan Heres

Germany

Katharin Hermenau

Germany

Morten Hesse

Denmark

Negoro Hideki

Japan

Christoph Hiemke

Germany

Matthew Hill

United Kingdom

Michele Hill

Ireland

Hubertus Himmerich

Germany

Kumazaki Hirokazu

Japan

Janet Hoenicka

Spain

Michael Höfler

Germany

Margaret Hogan

Tanzania

Eva Hogervorst

United Kingdom

Vera Hollen

USA

Kristina Holmgren

Sweden

Arne Holte

Norway

Paul Holtzheimer

USA

Anita Holzinger

Austria

David Hong

USA

Nicholas Horton

USA

Jonathan Houdmont

United Kingdom

Yingfen Hsia

United Kingdom

Klaas Huijbregts

Netherlands

Sarah Hunter

USA

Rene Hurlemann

Germany

Gerard Hutchinson

Trinidad And Tobago

Krista Huybrechts

USA

Thomas Hyphantis

Greece

Felice Iasevoli

Italy

Benjamin Iffland

Germany

Mari Ikeda

Japan

Masatoshi Inagaki

Japan

Masumi Inagaki

Japan

Waguih Ishak

USA

Yuko Ishizaki

Japan

K.S. Jacob

India

Claudia Jacova

Canada

Ben Jansen

USA

A Cecile Jw Janssens

Netherlands

Ignacio Jáuregui-Lobera

Spain

Rachel Jenkins

United Kingdom

Hong Jin Jeon

South Korea

Qing Jiao

China

Sian Jones

United Kingdom

Andreas Joos

Germany

Rikke Jørgensen

Denmark

Robert Joseph

USA

Mario Juruena

Brazil

Yoko Kamio

Japan

Thomas Kammer

Germany

Richard Antony Alexander Kanaan

United Kingdom

Gen Kanayama

USA

Yasuhiro Kaneda

Japan

Joshua Kantrowitz

USA

Nestor Damian Kapusta

Austria

Katherine Karlsgodt

USA

Kiyoto Kasai

Japan

Yuka Kato

Japan

Ines Kaufmann

Germany

Charlotte Keating

Australia

Robert Keeley

USA

Monika Keller

Germany

Fionn Kelly

Ireland

Harry Kennedy

Ireland

Michelle Kermode

Australia

Lars Kessing

Denmark

Ronald Kessler

USA

Margaret Keyes

USA

Alaptagin Khan

USA

Arifulla Khan

USA

Judi Kidger

United Kingdom

Martijn Kikkert

Netherlands

Melissa Kimber

Canada

Michael King

United Kingdom

Dawn Kingston

Canada

Bruce Kinon

USA

Patricia Kinser

USA

Eugene Kinyanda

Uganda

George Kirov

United Kingdom

Toshifumi Kishimoto

Japan

Toshinori Kitamura

Japan

Evan Kleiman

USA

Angela Klein

USA

Ryan Klein

Canada

Bonnie Klimes-Dougan

USA

Emily Klineberg

Australia

Stefan Kloiber

Germany

Sarah Knowles

United Kingdom

Brandon Kohrt

Nepal

Kaija-Leena Kolho

Finland

Tsuyoshi Kondo

Japan

George Konstantakopoulos

Greece

Anna Konstantellou

United Kingdom

Hans Koppeschaar

Netherlands

Jyrki Korkeila

Finland

Hirotaka Kosaka

Japan

Diana Koszycki

Canada

Kurt Kroenke

USA

Rune Kroken

Norway

Hans Kroon

Netherlands

Isabel Krug

Australia

Marina Kukla

USA

Hiroshi Kunugi

Japan

Poorna Kushalnagar

USA

Maria La Via

USA

Cornbelis Laban

Netherlands

Nicolai Ladegaard

Denmark

Beny Lafer

Brazil

Marueen Lage

USA

Daniel Lai

Canada

Meng-Chuan Lai

United Kingdom

Ratilal Lalloo

Australia

Jillian Lampert

USA

Glenn Landers

USA

Anne-Marit Langas

Norway

Jens M. Langosch

Germany

Yael Latzer

Israel

Alice Laudisio

Italy

Luca Lavagnino

Italy

David Lawrence

Australia

Joanna Leaviss

United Kingdom

Tania Lecomte

Canada

Bun-Hee Lee

South Korea

Hwa-Young Lee

South Korea

Moon-Soo Lee

USA

Sang-Hyuk Lee

South Korea

Stuart Lee

Australia

Verity Leeson

United Kingdom

Falk Leichsenring

Germany

Ann Lendrum

United Kingdom

Brian Leonard

Ireland

Bethany Leonhardt

USA

Martin Lepage

Canada

Yaakov Lerner

Israel

Siu-Wai Leung

Macao

Michael Levine

USA

Paul Lewandowski

Australia

Belinda Liddell

Australia

Elizabeth Liddle

United Kingdom

Roselind Lieb

Switzerland

Gregory Light

USA

Lisa Lilenfeld

USA

Pao-Yen Lin

Taiwan

Lene Lindberg

Sweden

Jean-Pierre Lindenmayer

USA

Paul S. Links

Canada

Garth Lipps

Jamaica

Jill Littrell

USA

James Livingston

Canada

Secundino López-PoUSA

Spain

Mario Louza

Brazil

Qing Lu

China

Lars-Gunnar Lundh

Sweden

Kien Hoa Ly

Sweden

Paul Lysaker

USA

Annmarie Macnamara

USA

Daniel Maixner

USA

Winnie Mak

Hong Kong

Dolores Malaspina

USA

Marcelo Mamede

Brazil

Katharina Manassis

Canada

Mirko Manchia

Italy

Vijaya Manicavasagar

Australia

Kristoffer Månsson

Sweden

Outi Mantere

Finland

Carlo Marchesi

Italy

Mary Margaretten

USA

Howard Margolese

Canada

Radboud Marijnissen

Netherlands

Kristian Markon

USA

David Marks

USA

Regina P. Markus

Brazil

Gary Martin

USA

Francesca Martino

Italy

Enrica Marzola

Italy

Radi Masri

USA

Davide Massidda

Italy

Aino Mattila

Finland

Shahrzad Mavandadi

USA

Fermin Mayoral

Spain

Eva Mazzotti

Italy

Patrick McCabe

USA

Rosemarie McCabe

United Kingdom

Shane McCarthy

USA

Laurie McCormick

USA

Brett McDermott

Australia

James McGough

USA

John McGrath

Australia

Alan McGuire

USA

Nicole Mclaughlin

USA

Hamish Mcleod

United Kingdom

Ann McNeill

United Kingdom

Nick Meader

United Kingdom

Judith Meijer

Netherlands

Marco Menchetti

Italy

Emily Mendenhall

USA

Douglas Mennin

USA

Tiago Mestre

Portugal

Stephen Metraux

USA

Didier Meulien

France

Thomas Daniel Meyer

United Kingdom

Amy Mezulis

USA

Patrick Michaels

USA

Ioannis Michopoulos

Greece

Jeremy Miles

USA

Jeremy Miles

United Kingdom

Brian Miller

USA

Regina Miranda

USA

Mansha Mirza

USA

Malgorzata Miszkurka

Canada

Nicholas Mitchell

Canada

Yoshinori Mitsuhashi

Japan

Kei Mizuno

Japan

Toshiki Mizuno

Japan

Shakeh Moamrtin

Australia

Markus Moessner

Germany

Jacqueline Mogle

USA

Mohabbat Mohseni

Iran

Chi Chiu Mok

Hong Kong

Roy Moncayo

Austria

Estrella Montoya

Netherlands

Kevin Morgan

United Kingdom

Norio Mori

Japan

Yusuke Moriguchi

Japan

Gunnar Morken

Norway

David Morris

USA

Derek Morris

Ireland

Richard Morriss

United Kingdom

Fedza Mujic

United Kingdom

Marco Mula

Italy

Kai Müller

Germany

Adrian Mundt

United Kingdom

Ricardo Munoz

USA

Mike Murphy

Ireland

Stuart Murray

Australia

Christiane Muth

Germany

Jochen Mutschler

Switzerland

Lori Nabors

USA

Akio Nakai

Japan

Motoaki Nakamura

Japan

Noeline Nakasujja

Uganda

Davide Nardo

Italy

Bruno Nazar

Brazil

David Ndetei

Kenya

Charles Nelson

USA

Jeffrey Newcorn

USA

Elizabeth Newnham

Australia

D. Jeffrey Newport

USA

Hong Ni

China

Charla Nich

USA

Angela Nickerson

Australia

Helmut Niederhofer

Germany

Inga Niedtfeld

Germany

Pentti Nieminen

Finland

Doris Nilsson

Sweden

Katsuhiko Nishimori

Japan

Margaret Niznikiewicz

USA

Willem Nolen

Netherlands

Greta Noordenbos

Netherlands

Tine Nordgreen

Norway

Carol North

USA

Roberto Nuevo

Spain

Paula Nunes

Brazil

John Nurnberger

USA

Desmond Oathes

USA

Kimberly O'Brien

USA

Rory O'Connor

United Kingdom

Stephen O'Connor

USA

Michel Odent

United Kingdom

Michael Gerhard Odenwald

Germany

Takashi Okada

Japan

Masayuki Okuda

Japan

Yasuyuki Okumura

Japan

Michele Okun

USA

Thomas Olino

USA

José Manuel Olivares

Spain

Mark Opler

USA

Killian O'Rourke

Ireland

Ragnhild Orstavik

Norway

Augustine Osman

USA

Eva Osório

Portugal

Soren Dinesen Ostergaard

Denmark

Margareta Östman

Sweden

Simon Øverland

Norway

Maciej Owecki

Poland

Christabel Owens

United Kingdom

Magdalena Paczkowski

USA

Chi-Un Pae

South Korea

Kathleen Pajer

Canada

Utpal Pajvani

USA

Ananda Pandurangi

USA

Tae Won Park

South Korea

George Parker

USA

Josef Parnas

Denmark

Timo Partonen

Finland

Augusto Pasini

Italy

Julie Patrick

USA

Elisabetta Patron

Italy

Scott Patten

Canada

Moli Paul

United Kingdom

Constança Paúl

Portugal

Carsten Bøcker Pedersen

Denmark

Per Bernhard Pedersen

Norway

Wenceslao Penate

Spain

David Penetar

USA

David Penn

USA

Marisol Perez

USA

Andrea Pfennig

Germany

Gianni Pirelli

USA

Valentino Pironti

United Kingdom

David Plante

USA

Graham Pluck

Japan

Michele Poletti

Italy

Maurizio Pompili

Italy

Alexander Ponizovsky

Israel

Raffaele Popolo

Italy

Maj-Britt Posserud

Norway

Nadine Potthast

Germany

Esther PoUSA Tomas

Spain

Michael Poyurovsky

Israel

Peter Pregelj

Slovenia

Antonio Preti

Italy

Amy Price

USA

Jonathan Price

United Kingdom

Stefan Priebe

United Kingdom

Jonathan Prince

USA

Elizabeth Pulgaron

USA

David Pulsford

United Kingdom

Rajeshwari Punekar

USA

Bernd Puschner

Germany

Norbert Quadflieg

Germany

Joaquim Radua

United Kingdom

Maria Augusta Raggi

Italy

Mathias Rask-Andersen

Sweden

Nicola Reavley

Australia

Warren Reich

USA

Siobhan Reilly

United Kingdom

Leslie Rescorla

USA

Wolfgang Retz

Germany

Valdo Ricca

Italy

Debra Rickwood

Australia

Matthias Riepe

Germany

Jamie Ringer

USA

Heleen Riper

Netherlands

Jennifer Robinson

USA

Jo Robinson

Australia

Nicolas Rohleder

USA

Irene Romera

Spain

Haydee Rosas-Vargas

Mexico

Jennifer Rose

USA

Kathryn Rose

USA

Isabelle Rouch

France

Kevin Rounding

Canada

Julia Rucklidge

New Zealand

Abraham Rudnick

Canada

Montserrat Rue

Spain

Giovanni Maria Ruggiero

Italy

M Angeles Ruiperez

Spain

Jeroen Ruwaard

Netherlands

Ned Sacktor

USA

John Sadler

USA

Cheryl Sadowski

Canada

Joseph Allan Sakdalan

New Zealand

Raimo K. R. Salokangas

Finland

Zainab Samaan

Canada

Carole Samango-Sprouse

USA

Cristina Sampaio

USA

Marsal Sanches

USA

Gabriele Sani

Italy

Paolo Santonastaso

Italy

Kionna Santos

Brazil

Alice Saperstein

USA

Anna Sasdelli

Italy

SUSAn Sawyer

Australia

Ingo Schäfer

Germany

Martin Schecklmann

Germany

Kolja Schiltz

Germany

Ben Schmand

Netherlands

Louis Schmidt

Canada

Stefanie Schmidt

Switzerland

Ulrike Schmidt

United Kingdom

Norbert Schmitz

Canada

Justine Schneider

United Kingdom

Kristin Schneider

USA

Carlos Schönfeldt-Lecuona

Germany

Didier Schrijvers

Belgium

Beate M. Schulze

Switzerland

Ulrich Schweiger

Germany

Soraya Seedat

South Africa

Cristina Segura-Garcia

Italy

Abdu Seid

Denmark

Annina Seiler

Switzerland

Dallas Seitz

Canada

Ana R. Sepulveda

Spain

Fernanda Serpeloni

Germany

Omar Sery

Czech Republic

Jonathan Shaffer

USA

Lokesh Shahani

USA

Chi-Yung Shang

Taiwan

Ann Sharpley

United Kingdom

Richard Shelton

USA

Tianmei Si

China

Margaret Sibley

USA

Larry Siever

USA

Cecilia Sighinolfi

Italy

Alan Simpson

United Kingdom

Sandy Simpson

Canada

Swaran Preet Singh

United Kingdom

Petros Skapinakis

Greece

David Slattery

Germany

Ladislav Slovacek

Czech Republic

Hugo Matthias Smeets

Netherlands

Andy Smith

United Kingdom

Anna Smith

United Kingdom

Daniel Smith

United Kingdom

Patrick Smith

USA

Shubulade Smith

United Kingdom

Anne Söderlund

Sweden

Nerissa Soh

Australia

Nadia Solowij

Australia

Iris Sommer

Netherlands

Cesar Soutullo

Spain

Julia Friederike Sowislo

Switzerland

Michael Soyka

Switzerland

Gianfranco Spalletta

Italy

Mario Speranza

France

Glen Spielmans

USA

James Spira

USA

Matthew Spittal

Australia

Andria Spyridou

Msc.

Vladan Starcevic

Australia

Maria Steenkamp

USA

Mark Stein

USA

Robert Stephens

USA

Richard Stevenson

Australia

Heidi Stöckl

United Kingdom

Martin Strassnig

USA

Kelcey Stratton

USA

Zbigniew Struzik

Japan

Mythily Subramaniam

Singapore

Philip Sugarman

United Kingdom

Danny Sullivan

Australia

Matthew Sunderland

Australia

Etsuji Suzuki

Japan

Takefumi Suzuki

Japan

Igor Svab

Slovenia

Elisabeth Svensson

Sweden

Wendy Swift

Australia

Andrei Szoke

France

Juan Sztajzel

Switzerland

Eskinder Tafesse

USA

Lubna Tahtamouni

Jordan

Qingrong Tan

China

Kiwamu Tanaka

Japan

Masaaki Tanaka

Japan

Rajiv Tandon

USA

Yanqing Tang

China

Tetsuya Tanioka

Japan

Linda Tapsell

Australia

Steven Targum

USA

Ilaria Tarricone

Italy

Kuowei Tay

Australia

Daniel Taylor

USA

David Taylor

United Kingdom

Kate Tchanturia

United Kingdom

Martin Teicher

USA

Martina Teichert

Netherlands

David Terburg

Netherlands

Martin Teufel

Germany

Cornelia Thiels

Germany

Sanjeev Thomas

India

Per Hove Thomsen

Denmark

Sannie Vester Thorsen

Denmark

Birgitte Thylstrup

Denmark

Henning Tiemeier

Netherlands

Liu Tieqiao

China

Maria Tillfors

Sweden

Christine Timko

USA

Arun Tiwari

Canada

Patrizia Todisco

Italy

Barry Tolchard

Australia

Mitsunao Tomioka

Japan

Akemi Tomoda

Japan

Ingrid Tonhajzerova

Slovakia

Mattie Tops

Netherlands

Michelle Torok

Australia

Ivan Torres

Canada

Walter Torres

USA

E Fuller Torrey

USA

William Torrey

USA

Derek Tracy

United Kingdom

Tom Trauer

Australia

Ian Treasaden

United Kingdom

Jitendra Trivedi

India

Mehul Trivedi

USA

Alfonso Troisi

Italy

Do Tromp

USA

Nicholas Troop

United Kingdom

Miao-Yu Tsai

Taiwan

Samson Tse

Hong Kong

Wan-Ling Tseng

USA

Kenji J. Tsuchiya

Japan

Daniel Turner

Germany

Golfo Tzilos

USA

Hiroyuki Uchida

Japan

Alp Ucok

Turkey

Masayo Uji

Japan

Zsolt Unoka

Hungary

Himanshu Upadhyaya

USA

Jason Uslaner

USA

Michael Ussher

United Kingdom

Geurt Van De Glind

Netherlands

Geertje Van De Ven

Netherlands

Marjan Van Den Akker

Netherlands

Erik Van Duijn

Netherlands

Andreas Van Egmond-Fröhlich

Austria

Jan Van Hecke

Belgium

Trevor Van Mielro

Canada

Davy Vancampfort

Belgium

Ryan Vandrey

USA

Helen-Maria Vasiliadis

Canada

Irene Vavasour

Canada

Maria Vazquez-Montes

United Kingdom

Ganesan Venkatasubramanian

India

Peter Ventevogel

Netherlands

Marjolein Verhoeven

Netherlands

Henk Verloo

Switzerland

Benjamin Vicente

Chile

Andelko Vidovic

Croatia

Giovanni Viscogliosi

Italy

Michael Vitacco

USA

Benedetto Vitiello

USA

Mirela Vlastelica

Croatia

Silja Vocks

Germany

Jen Vohs

USA

Corrine Voils

USA

Jan Volavka

USA

Martin Voracek

Austria

Lakshmi Voruganti

Canada

Benjamin Vyssoki

Austria

Tracey Wade

Australia

Angela Wagner

USA

Jerome Wakefield

USA

Fredrik A. Walby

Norway

Michael Waller

Australia

Kate Walsh

USA

Sophie Walsh

Israel

Martin Walter

Germany

Sebastian Walther

Switzerland

Bruce Wampold

USA

Johannes Wancata

Austria

Liang-Jen Wang

Taiwan

Wei Wang

China

Yuan-Pang Wang

Brazil

Virginia Warner

USA

Napoleon Waszkiewicz

Poland

Delma-Jean Watts

USA

Lesley Jo Weaver

USA

Jeremiah Weinstock

USA

Abraham Weizman

Israel

Nomi Werbeloff

Israel

Shirli Werner

Israel

Lars Westberg

USA

Rhiannon Whitaker

United Kingdom

Julie Whitney

United Kingdom

Sarah Wilker

Germany

Christopher Williams

United Kingdom

Thomas Wise

USA

Albert Wong

Canada

Hans Wouters

Netherlands

Chung-Hsuen Wu

Taiwan

Renrong Wu

China

Xia Wu

China

Katharina Wulff

United Kingdom

Jonathan Wynn

USA

Rolf Wynn

Norway

Jian Xu

China

Weili Xu

Sweden

Atsurou Yamada

Japan

Yushiro Yamashita

Japan

Yen Kuang Yang

Taiwan

Philip Yanos

USA

Shuqiao Yao

China

Chiho Yatsuga

Japan

Cheng-Fang Yen

Taiwan

Simon Yolung

Canada

Shachar Yonai

Israel

SUSAn Young

United Kingdom

Nicole Yuan

USA

Muhamad Saiful Bahri Yusoff

Malaysia

Benjamin Zablotsky

USA

Peter Zachar

USA

Pawel Zagozdzon

Poland

Clement Zai

Canada

Clement Zai

Canada

Yu-Feng Zang

China

Sergio Zanini

Italy

Leonardo Zaninotto

Italy

Douglas Zatzick

USA

Patrizia Zeppegno

Italy

Jie Zhang

USA

Xiang-Yang Zhang

USA

Xiuwu Zhang

USA

Bonnie Zima

USA

Mathias Zink

Germany

Stephan Zipfel

Germany

Yaara Zisman-Ilani

Israel

## Pre-publication history

The pre-publication history for this paper can be accessed here:

http://www.biomedcentral.com/1471-244X/14/40/prepub

